# Access to employment: A comparison of autistic, neurodivergent and neurotypical adults’ experiences of hiring processes in the United Kingdom

**DOI:** 10.1177/13623613221145377

**Published:** 2023-01-04

**Authors:** Jade Davies, Brett Heasman, Adam Livesey, Amy Walker, Elizabeth Pellicano, Anna Remington

**Affiliations:** 1 University College London, UK; 2 York St John University, UK; 3 Neurodiversity Works, UK; 4 Macquarie University, Australia

**Keywords:** adulthood, autism, employment, recruitment

## Abstract

Autistic people face high unemployment rates. One reason for this may be that hiring processes are inaccessible. This study aimed to establish autistic people’s unique experiences of hiring processes in the United Kingdom, by comparing them to the experiences of non-autistic neurodivergent people and neurotypical people. Using qualitative and quantitative data from 225 autistic, 64 non-autistic neurodivergent and 88 neurotypical adults, we identified a series of (dis)similarities in participants’ views and experiences of recruitment for employment. Similarities across the three groups included (1) frustration with the focus on social skills; (2) a perceived need for more flexible hiring processes; (3) a desire for more clarity and (4) the importance of the environment. Participants also acknowledged the important role employers play in one’s decision to disclose a diagnosis or access need. Yet, autistic people faced a set of unique barriers to successful recruitment, over and above those that non-autistic people faced. For example, the perceived pressure to mask autistic traits to succeed and concerns about stigma and discrimination. Participants’ recommendations for improvements included the use of more practical recruitment strategies (e.g. work trials), more clarity about what to expect, and improvements in recruiters’ understanding of the challenges autistic and neurodivergent candidates may face.

**Lay abstract:**

Autistic people are less likely to have a job than non-autistic people. One reason for this may be that hiring processes (e.g. job applications, interviews) can be challenging for autistic people. To better understand the experiences of hiring processes in the United Kingdom, we asked 225 autistic, 64 neurodivergent (but not autistic) and 64 adults with no reported area of neurodivergence questions about their experiences using an online survey. We found a range of similarities and differences in responses. For example, participants in all three groups were frustrated with the focus on social skills in recruitment and said they wanted more practical methods (e.g. work trials) that help them show their skills and abilities. Autistic and otherwise neurodivergent participants discussed the importance of the environment (e.g. the interview/assessment room) in improving experiences. Participants also discussed how employers can impact whether somebody decides to disclose their diagnosis or needs – or not. Autistic people experienced some barriers to successful recruitment that non-autistic people did not. For example, autistic people felt they had to hide their autistic traits to gain employment and many autistic people were worried about being discriminated against if they disclosed that they were autistic during the hiring process. To make experiences better, our participants said that employers should offer candidates different recruitment methods and give them more information about the hiring process. They also said employers should improve their understanding of autism and other hidden disabilities so they know the challenges that people might face during recruitment.

Employment is important for a person’s well-being (Clark & Lepinteur, 2019; Paul & Moser, 2009) and economic gain for individuals and broader society. Autistic people are no different: research shows that employment can positively impact autistic individuals’ mental health, well-being and quality of life (Mason et al., 2018; Roux et al., 2015; Walsh et al., 2014), particularly when employment is supported (García-Villamisar & Hughes, 2007). Conversely, unemployment and job loss are associated with higher depressive symptoms and lower overall quality of life for autistic people (Renty & Roeyers, 2006; Taylor et al., 2021). The specific nature of the relationship between employment and mental health is unclear – although poorer mental health and life satisfaction in autistic adults, relative to non-autistic adults, have been shown to be at least partially explained by a greater vulnerability to negative life experiences, such as unemployment or malemployment (Griffiths et al., 2019). Securing and maintaining employment might therefore be one important factor in improving mental health outcomes in autistic adults.

Yet, 80% of autistic people are estimated to be unemployed worldwide (Ki-moon, 2015) and unemployment rates in the United Kingdom are higher for autistic people than other disability groups (Office for National Statistics, 2021). This is despite many autistic people being willing and able to engage in employment (Hendricks, 2010) and possessing a range of unique skills and qualities that may be of particular value to employers, for example, attention to detail, reliability and a tolerance for repetition (Austin & Pisano, 2017; Cope & Remington, 2021; Ortiz, 2020; Russell et al., 2019; Scott et al., 2017). Indeed, organisations with autistic employees have emphasised the wide range of benefits that neurodiversity^1^ can bring to employment. For example, SAP software solutions (a multinational software corporation and prominent employer of neurodivergent people) report a direct link between workplace diversity and innovation, with innovations from their neurodivergent^2^ employees contributing to savings of approximately US$40 million (Fox, 2020). Similarly, Hewlett Packard Enterprise reports their neurodivergent employees to be up to 30% more productive than their neurotypical^3^ counterparts (Nelson, 2018).

Given the meaningful contributions that autistic people can make in the workplace, and the gap between the rates of those who want to work and those who are employed, it is clear that many organisations are missing out on the talent and skills that autistic people can bring to their workforce. One reason for this disconnect may be that inaccessible hiring processes (also referred to as recruitment processes) act as a barrier to autistic people obtaining employment (Vincent, 2020). The current study therefore sought to examine the recruitment experiences of autistic people, establish to what extent autistic people’s experiences differ from those of otherwise neurodivergent, and neurotypical people, and identify specific areas in which typical hiring processes could be improved.

Autistic people are likely to face several hurdles during the hiring process. First, barriers exist in finding suitable employment opportunities. Indeed, the process of hearing about a job and deciding to apply can be – albeit unwittingly – biased against autistic people. For example, a large proportion of jobs are secured through existing social connections (Markel & Elia, 2016). Yet, autistic people tend to have smaller social networks (Orsmond et al., 2004, 2013) and, as such, may struggle finding appropriate employment. Even when jobs are shared widely, many employers repackage existing vacancies using generic job descriptions that prioritise generic ‘baseline skills’ such as ‘team-working’ or ‘communication’ skills as opposed to ‘specialised skills’ that are specific and relevant to the job role (Hurley-Hanson et al., 2020). This could be problematic for autistic people who are likely to interpret language literally (Walenski et al., 2006) and may therefore not apply for a job if they feel they do not entirely fulfil the specific criteria set out as required for the role (Nagib & Wilton, 2020; Vincent, 2020). Indeed, many autistic people face challenges in social communication (American Psychiatric Association, 2013) and may therefore be discouraged from applying for roles that require high levels of communication skills. This potential reluctance for autistic people to apply for roles in which they do not fulfil all the job criteria is distinct to their non-autistic counterparts who are likely to apply for job roles despite only broadly fulfilling the criteria provided in the job description (Markel & Elia, 2016).

Second, autistic people are likely to face additional barriers during the initial written job application. For example, autistic people are less likely to be offered work experience opportunities and may experience challenges in tailoring the experience they *do* have to the requirements of the prospective job role (Baldwin et al., 2014; Graetz, 2010; Vincent, 2020; Wilczynski et al., 2013). As such, autistic candidates may struggle to showcase their skillset to potential employers. Indeed, evidence from a recent white paper suggests that the increasing use of artificial intelligence in recruitment (e.g. curriculum vitae screeners) is problematic in this regard as systems are unable to account for such individual differences in experiences (Nugent et al., 2020). As a result, autistic candidates may be likely to be ‘screened out’ before they are able to demonstrate their skills.

Third, specific barriers pertaining to the employment interview exist (e.g. Sarrett, 2017). The employment interview is one of the most common recruitment devices used by organisations (Levashina et al., 2014; Macan, 2009; Wilk & Cappelli, 2003) and successful performance is often contingent on a series of interpersonal communication skills, such as the effective use of verbal and non-verbal communication, presentation skills and impression management (Bourdage et al., 2020; DeGroot & Motowidlo, 1999; Lorenz et al., 2016; Macan, 2009; Peck & Levashina, 2017; Van Iddekinge et al., 2007). However, there is evidence to suggest that autistic people may be more likely to struggle in this regard than non-autistic people, even if they are capable of doing the job in question. For example, some autistic people experience challenges in managing social expectations, understanding and engaging in verbal and non-verbal communication, and responding to interview questions that require an element of impression management (e.g. about one’s weaknesses) (Black et al., 2019; Flower, Hedley et al., 2019; Hendricks, 2010; Sarrett, 2017). Consequently, autistic candidates may be more likely than non-autistic candidates to struggle in employment interviews.

Additional barriers related to the employment interview exist. For example, interviewers often use open questions to probe about specific personal experiences (e.g. tell me about a time . . .), yet research shows that autistic people can experience difficulties in recalling episodic memories (Crane et al., 2009) and memory often declines the more open-ended task (Gaigg & Bowler, 2018). Indeed, autistic people often report needing additional processing time to make sense of what is being asked of them (Honeybourne, 2019). Yet, given the time constraints that employment interviews are often subjected to, this processing time is unlikely to be accounted for, potentially placing autistic people at a disadvantage. Furthermore, evidence suggests that employers, and therefore interviewers, often lack an understanding about autism and the specific challenges that autistic people may face (López & Keenan, 2014). As such, it is possible that many interviewers are unaware of the potential adjustments that could be implemented to support autistic candidates during recruitment. It is perhaps therefore unsurprising that employers typically show a preference towards employing non-autistic candidates over autistic candidates (Ameri et al., 2018; Flower, Dickens & Hedley, 2019).

Fourth, there are also practical barriers to the hiring process. In the first instance, attending an employment interview requires individuals to deviate from their daily routine. Given that autistic people often demonstrate a need for structure and routine and as such experience anxiety in unfamiliar settings (e.g. Milton & Sims, 2016), this deviation from daily life is likely to create anxiety and uncertainty surrounding the employment interview. Furthermore, many autistic people experience sensory sensitivities (Crane & Goddard, 2008; Tavassoli et al., 2014), including sensitivities to certain sights and sounds, and without appropriate adjustments, the interview environment can be overwhelming (Vincent, 2020).

As highlighted, there are good empirical reasons to expect that autistic people are likely to experience a unique set of challenges during the hiring process, over and above those that non-autistic people face. Yet, to our knowledge, no research has directly compared the experiences of autistic and non-autistic people during recruitment. It is therefore not yet clear precisely which challenges are common across all candidates and which challenges, if any, are specific to autistic people. This study aims to address this gap by comparing the first-hand perspectives of autistic, non-autistic neurodivergent and neurotypical (i.e. people with no disclosed area of neurodivergence) adults in the United Kingdom. We conclude by establishing ways in which organisations can adapt their processes to support autistic people, and the wider workforce, to access employment.

## Method

The current study forms part of a broader body of research examining autistic adults’ experiences of employment in the United Kingdom, using the Diverse Minds Survey. The Diverse Minds Survey was developed in collaboration with a group of autistic reviewers and was hosted online, powered by Qualtrics. The survey gathers information about an individual’s neurodiversity and their employment experiences, including optional modules on specific aspects of employment, including recruitment and hiring processes. While efforts were made to make the survey as inclusive as possible (e.g. including options to adjust the screen colour and contrast, and using lay-person language throughout), participants were nevertheless required to complete an in-depth survey involving reflecting on, and discussing, their personal experiences. As such, we acknowledge that the survey likely precluded the involvement of adults with intellectual disability.

The Diverse Minds Survey was advertised through (1) Autistica’s Discover Network for autistic people interested in taking part in research; (2) the research team’s publicly accessible social media channels and (3) organisations linked to the project that had expressed an interest in understanding more about neurodiversity and employment. The current study examined participants’ responses to questions regarding experiences of recruitment between March 2019 and April 2020.^4^

### Participants

Participants were all aged above 18 years and had experience of hiring processes in the United Kingdom. The sample was originally intended to be divided into two groups: autistic participants and non-autistic participants. However, given the number of participants who had identified themselves as neurodivergent (including those who had a formal or self-diagnosis of a neurodevelopmental condition, other than autism, or a mental health condition), we took the opportunity to examine potential differences between: (1) autistic participants (including formally diagnosed and self-identified autistic people^5^), (2) non-autistic neurodivergent participants (including those who had a formal or self-diagnosis of a neurodivergent condition excluding autism, or a mental health condition) and (3) neurotypical participants (those without an identified neurodivergence). By April 2020, 241 autistic people, 83 non-autistic neurodivergent people and 113 neurotypical people had navigated to the recruitment survey. Of those, 16 autistic (6.6%), 19 non-autistic neurodivergent (22.9%) and 25 neurotypical (22.1%) participants were removed from analyses as they did not complete at least 50% of the recruitment-specific questions. In total, 225 autistic, 64 non-autistic neurodivergent and 88 neurotypical adults were included in the final sample.

The majority of autistic participants had a formal autism diagnosis (*n* = 192, 85.3%), with the remainder (*n* = 33, 14.7%) self-identifying as autistic. More than two thirds of autistic participants (*n* = 157, 69.8%) also disclosed a mental health condition. The most common diagnoses within the non-autistic neurodivergent sample included anxiety (*n* = 20, 31.3%), depression (*n* = 15, 23.4%) and dyslexia (*n* = 10, 15.6%). Approximately half of the neurodivergent (*n* = 27, 42.2%) and neurotypical participants (*n* = 48, 54.5%) identified as male, compared to less than 30% of the autistic participants (*n* = 64, 28.4%). Across the whole sample, there was a notable lack of diversity in regard to ethnicity, with the majority of participants in each group being from a white ethnic background (see Table 1). Similarly, the majority of participants in all three groups were educated to at least a bachelor’s degree level, and approximately one third of the neurotypical sample (*n* = 29, 33.0%) reported being in senior-level employment.

**Table 1.** Demographic characteristics.

Background variables	Autistic participants (*n* = 225) ^a^	Non-autistic neurodivergent participants (*n* = 64)	Neurotypical participants (*n* = 88)	Group comparisons^b^
Gender identity				A > ND****, NT* **ND = NT**
Female (including transwoman)	145 (64.4%)	35 (54.7%)	40 (45.5%)	
Male (including transman)	64 (28.4%)	27 (42.2%)	48 (54.5%)	
Non-binary	12 (5.3%)	0 (0.0%)	0 (0.0%)	
Other	4 (1.8%)	0 (0.0%)	0 (0.0%)	
Prefer not to say	0 (0.0%)	2 (3.1%)	0 (0.0%)	
Age (years)				**A = ND = NT**
18–25	26 (11.6%)	5 (7.8%)	10 (11.4%)	
26–35	51 (22.7%)	23 (35.9%)	23 (26.1%)	
36–45	55 (24.4%)	13 (20.3%)	20 (22.7%)	
46–55	67 (29.8%)	16 (25.0%)	29 (33.0%)	
56–65	24 (10.7%)	6 (9.4%)	4 (4.5%)	
66–75	2 (0.9%)	1 (1.6%)	2 (2.3%)	
Ethnicity^c^				A < NT*, **ND = A, NT**
White	161 (71.6%)	56 (87.5%)	78 (88.6%)	
Black/African/Caribbean/Black British	0 (0.0%)	0 (0.0%)	1 (1.1%)	
Asian/Asian British	0 (0.0%)	1 (1.6%)	4 (4.5%)	
British/English/Scottish/United Kingdom	24 (10.7%)	2 (3.1%)	2 (2.3%)	
Mixed/multiple ethnic groups	10 (4.4%)	1 (1.6%)	1 (1.1%)	
Eastern European	2 (0.9%)	0 (0.0%)	0 (0.0%)	
South African	2 (0.9%)	0 (0.0%)	0 (0.0%)	
Ashkenazi Jewish	1 (0.4%)	0 (0.0%)	0 (0.0%)	
Latin	1 (0.4%)	0 (0.0%)	0 (0.0%)	
Australian	1 (0.4%)	0 (0.0%)	0 (0.0%)	
Irish	0 (0.0%)	1 (1.6%)	0 (0.0%)	
Indian	0 (0.0%)	0 (0.0%)	1 (1.1%)	
Undisclosed	23 (10.2%)	3 (4.7%)	1 (1.1%)	
Highest level of education				**A = ND = NT**
Bachelor’s degree (e.g., Bsc, BA, BEd)	63 (28.0%)	22 (34.4%)	28 (31.8%)	
Masters degree (e.g., MA, MSc, MEd)	58 (25.8%)	19 (29.7%)	39 (44.3%)	
Vocational qualification (e.g., BTEC, GNVQ, HND)	23 (10.2%)	5 (7.8%)	1 (1.1%)	
A/AS level^d^	23 (10.2%)	3 (4.7%)	4 (4.5%)	
Other postgraduate study (e.g., PGCe, PGDip)	18 (8.0%)	4 (6.3%)	6 (6.8%)	
Doctorate	16 (7.1%)	3 (4.7%)	5 (5.7%)	
GCSE’s^e^	12 (5.3%)	4 (6.3%)	3 (3.4%)	
Foundation degree	6 (2.7%)	3 (4.7%)	0 (0.0%)	
No formal qualifications	4 (1.8%)	1 (1.6%)	0 (0.0%)	
Other (e.g. fellowship to professional body)	2 (0.9%)	0 (0.0%)	2 (2.3%)	
Employment status				A < ND*, NT*; **ND = NT**
Employed full-time	86 (38.2%)	48 (75.0%)	73 (83.0%)	
Employed part-time	51 (22.7%)	9 (14.1%)	13 (14.8%)	
Self-employed	20 (8.9%)	1 (1.6%)	1 (1.1%)	
Unemployed (looking for work)	19 (8.4%)	2 (3.1%)	0 (0.0%)	
Unemployed (not looking for work)	16 (7.1%)	1 (1.6%)	0 (0.0%)	
Student	10 (4.4%)	1 (1.6%)	1 (1.1%)	
Volunteer	6 (2.7%)	0 (0.0%)	0 (0.0%)	
Full-time career	6 (2.7%)	0 (0.0%)	0 (0.0%)	
Other	6 (2.7%)	0 (0.0%)	0 (0.0%)	
Retired	5 (2.2%)	2 (3.1%)	0 (0.0%)	
Satisfaction with job role				A < ND**, NT*; ND < NT*
Satisfied	99 (44.0%)	40 (62.5%)	80 (90.9%)	
Dissatisfied	66 (29.3%)	11 (17.2%)	1 (1.1%)	
Uncertain	46 (20.4%)	8 (12.5%)	5 (5.7%)	
Neither satisfied nor unsatisfied	6 (2.7%)	0 (0.0%)	0 (0.0%)	
Other	5 (2.2%)	0 (0.0%)	0 (0.0%)	
N/A	3 (1.3%)	2 (3.1%)	0 (0.0%)	
Prefer not to say	0 (0.0%)	3 (4.7%)	1 (1.1%)	
Number of past employers				A > NT*; **ND = NT, A**
None	5 (2.2%)	4 (6.3%)	3 (3.4%)	
1–2	22 (9.8%)	13 (20.3%)	27 (30.7%)	
2–4	49 (21.8%)	16 (25.0%)	31 (35.2%)	
4–6	37 (16.4%)	12 (18.8%)	11 (12.5%)	
>6	110 (48.9%)	19 (29.7%)	16 (18.2%)	
Prefer not to say	2 (0.9%)	0 (0.0%)	0 (0.0%)	
Most recent income (£)				A < ND*, NT*; ND < NT***
<10,000	45 (20.0%)	6 (9.4%)	0 (0.0%)	
10,000–19,999	59 (26.2%)	6 (9.4%)	3 (3.4%)	
20,000–29,999	51 (22.7%)	11 (17.2%)	6 (6.8%)	
30,000–39,999	27 (12.0%)	13 (20.3%)	24 (27.3%)	
40,000–49,999	9 (4.0%)	13 (20.3%)	11 (12.5%)	
50,000–59,999	7 (3.1%)	2 (3.1%)	6 (6.8%)	
60,000–79,999	7 (3.1%)	7 (10.9%)	12 (13.6%)	
80,000–99,999	3 (1.3%)	2 (3.1%)	10 (11.4%)	
100,000–149,999	4 (1.8%)	1 (1.6%)	9 (10.2%)	
>150,000	0 (0.0%)	0 (0.0%)	1 (1.1%)	
Prefer not to say	13 (5.8%)	3 (4.7%)	6 (6.8%)	
Highest level worked at				**A = ND = NT**
Mid-level employment	94 (41.8%)	32 (50.0%)	39 (44.3%)	
Entry level/graduate employment	66 (29.3%)	14 (21.9%)	18 (20.5%)	
Senior-level employment	39 (17.3%)	11 (17.2%)	29 (33.0%)	
Intern, apprentice or volunteer	14 (6.2%)	4 (6.3%)	1 (1.1%)	
Other	7 (3.1%)	1 (1.6%)	0 (0.0%)	
Prefer not to say	5 (2.2%)	2 (3.1%)	1 (1.1%)	
Sector (top three for each group shown)				
Education	41 (18.2%)	4 (6.3%)	3 (3.4%)	
Healthcare	29 (12.9%)	3 (4.7%)	0 (0.0%)	
Public sector	22 (9.8%)	1 (1.6%)	0 (0.0%)	
Technology	8 (3.6%)	12 (18.8%)	18 (20.5%)	
Transport	1 (0.4%)	11 (17.2%)	15 (17.0%)	
Infrastructure	3 (1.3%)	8 (12.5%)	21 (23.9%)	

a There were no significant differences in the demographic information between formally diagnosed and self-diagnosed autistic participants.

b A = autistic, ND = neurodivergent, NT = neurotypical.

c The question concerning ethnicity had a free-text response box. Some participants disclosed their nationality as opposed to their ethnicity.

d A/AS Levels are qualifications in the United Kingdom, typically taken between 16 and 18 years of age.

e GCSEs are qualifications in the United Kingdom, typically taken between 14 and 16 years of age. *p < 0.001, **p = 0.002, ***p = 0.003, ****p = 0.006.

Fisher’s exact tests employing a Bonferroni correction for multiple comparisons were conducted to determine if there were group differences in participant characteristics. There were significantly more females in the autistic group (*n* = 145, 64.46%) than the non-autistic neurodivergent (*n* = 35, 54.7%; *p* = 0.006) and neurotypical (*n* = 40, 45.5%; *p* < 0.001) groups. The majority of participants in all three groups were in a form of paid employment, including full-time employment, part-time employment and self-employment. However, significantly more neurotypical (*n* = 73, 83.0%; *p* < 0.001) and non-autistic neurodivergent (*n* = 48, 75.0%; *p* < 0.001) participants were in full-time employment, relative to autistic participants (*n* = 86, 38.2%). Similarly, there were significant group differences in income, with more neurotypical participants’ salary being in a higher range than autistic (*p* < 0.001) and non-autistic neurodivergent (*p* = 0.003) participants. Non-autistic neurodivergent participants also reported having higher earning power than autistic participants (*p* < 0.001). Perhaps relatedly, significantly fewer autistic participants reported being satisfied with their current job role than non-autistic neurodivergent (*p* = 0.002) and neurotypical (*p* < 0.001) participants, and fewer non-autistic neurodivergent participants reported being satisfied than neurotypical participants (*p* < 0.001). See Table 1 for further demographic information and group comparisons.

### Materials

All participants completed the demographics module on the Diverse Minds Survey, including questions regarding their gender identity, ethnicity and highest level of education. The demographics module also contained questions concerning participants’ employment experiences (e.g. current employment status, satisfaction with current job role, sector of their most recent employer, highest level they had worked at, most recent income and number of past employers). The participants in the current study also completed a module regarding their experiences of recruitment processes. This module began by providing the following definition of recruitment: ‘recruitment processes include all the steps from a job being advertised to being informed about the outcome of the final assessment or interview’. Participants were then asked to select the recruitment techniques they had experienced from a series of predetermined options, such as ‘online test’, ‘psychometric test’ and ‘interview’, or provide examples of alternative recruitment experiences. Participants were also asked to reflect ‘how positively’ they would rate their experiences of each recruitment technique on a four-point Likert-type scale ranging from 1 (*very negative*) to 4 (*very positive*). Next, participants were asked a closed question regarding whether they had been able to provide feedback to employers about their recruitment experience. The module ended by asking participants two open questions probing for information related to examples of particularly positive and negative experiences, and how recruitment processes could be improved (see Supplementary Materials 1 for full survey).

### Procedure

The module regarding recruitment experiences took approximately 10 min to complete. Ethical approval was obtained through the Research Ethics Committee at UCL Institute of Education, Faculty of Education and Society (REC1149) and all participants gave informed consent to take part prior to participation.

### Data analysis

Quantitative data were analysed using IBM SPSS Statistics (Version 25; IBM Corp, 2017). Chi-square tests of independence and independent *t*-tests demonstrated that there was no significant difference between the quantitative responses from self-diagnosed and formally diagnosed autistic participants. As such, responses from all participants in the autistic group were considered together. Chi-square tests of independence (or, where relevant, Fisher’s exact test) were used to compare the distribution of responses to the closed questions between the three groups (autistic, non-autistic neurodivergent and neurotypical). Finally, one-way ANOVAs or, where necessary, Kruskal–Wallis tests examined group differences on the mean ratings of the recruitment methods.

Responses to the two open-ended questions were analysed using reflexive thematic analysis (Braun & Clarke, 2006, 2013, 2019) Data were analysed within a critical realist framework, involving the inductive (bottom-up) identification of semantic meanings in the data (Braun & Clarke, 2013). Data analysis was driven by J.D., a researcher with expertise in autism research, who recursively proceeded through the stages of data familiarisation, inductive coding, theme development and review. With support from A.R., a senior researcher with expertise in autism research, J.D. reviewed the results, refining where necessary, to establish a final set of comprehensive and distinct themes and subthemes. All authors, a neurodiverse group consisting of autistic, neurodivergent and neurotypical individuals, agreed on the final set of themes. Responses from autistic participants (including both formally diagnosed and self-diagnosed) were analysed independently from the responses from non-autistic neurodivergent and neurotypical participants. The broader themes that were developed were inspected to ensure that they were representative of both self-diagnosed and formally diagnosed autistic participants’ experiences.

### Community involvement

Two autistic co-authors (A.L. and A.W.) were involved in the development of the questionnaire, interpreting the findings, providing feedback on drafts of the article and developing subsequent recommendations for those involved in hiring processes.

## Results

### Quantitative results

#### Experiences of recruitment methods

In total, 202 autistic participants, 61 non-autistic neurodivergent and 86 neurotypical participants gave details regarding the recruitment methods they had experienced (see Table 2). The interview was the most common recruitment method experienced, with the vast majority of autistic (*n* = 199, 98.5%) and neurotypical participants (*n* = 83, 96.5%), and all non-autistic neurodivergent participants (*n* = 61, 100%), reporting that they had taken part in an employment interview. Also common were online tests (52.2% autistic participants, 55.7% non-autistic neurodivergent participants and 55.8% neurotypical participants) and group tasks (47.5% autistic participants, 47.5% non-autistic neurodivergent participants and 50% neurotypical participants). In open-text responses, participants also offered insight into a range of alternative hiring processes that had been experienced. These responses were categorised into skills assessments, presentations, role play activities and informal discussions for the purpose of analysis.

**Table 2.** Recruitment methods experienced by autistic and non-autistic participants (*n* = 349).

	Autistic participants (*n* = 202)	Non-autistic neurodivergent participants (*n* = 61)	Neurotypical participants (*n* = 86)	Group comparisons
Interview	199 (98.5%)	61 (100%)	83 (96.5%)	A = ND = NT
Written questionnaire	124 (61.4%)	30 (49.2%)	31 (36.0%)	A > NT*; ND = NT, A
Online test	106 (52.5%)	34 (55.7%)	48 (55.8%)	A = ND = NT
Group task	96 (47.5%)	29 (47.5%)	43 (50.0%)	A = ND = NT
Psychometric test	85 (42.1%)	32 (52.5%)	55 (64.0%)	NT > A*; ND = NT, A
Work trial	71 (35.1%)	17 (27.9%)	16 (18.6%)	A = ND = NT
Skills assessments	21 (10.4%)	0 (0.0%)	3 (3.5%)	A = ND = NT
Presentation	8 (4.0%)	2 (3.3%)	2 (2.3%)	A = ND = NT
Role play activities	2 (1.0%)	0 (0.0%)	1 (1.2%)	A = ND = NT
Informal discussion	1 (0.5%)	0 (0.0%)	0 (0.0%)	A = ND = NT

Total percentages exceed 100% as the different recruitment methods were not mutually exclusive (i.e. participants could report on all of the recruitment methods they had experienced); *p < 0.001.

Chi-square tests of independence using a Bonferroni correction for multiple comparisons showed that significantly more autistic participants reported experiencing written questionnaires than neurotypical participants: χ^2^(1, *n* = 287) = 14.35, *p* < 0.001. Significantly, more neurotypical participants reported experiencing psychometric tests than autistic participants: χ^2^(1, *n* = 287) = 12.30, *p* < 0.001. No other group differences reached significance.

#### Perceptions of recruitment methods

Despite being the most commonly experienced recruitment method, interviews were not well endorsed by autistic people (*M*_rating_ = 2.21, *SD* = 0.78) (see Table 3). Group tasks were also perceived as particularly negative by autistic participants (*M*_rating_ = 1.60, *SD* = 0.80). One-way ANOVAs showed significant group differences in the ratings of interviews (*F*(2, 355) = 45.38, *p* < 0.001) and group tasks (*F*(2, 180) = 32.80, *p* < 0.001). Tukey’s post hoc tests revealed that autistic participants rated their experience of interviews and group tasks as significantly less positive than neurotypical (*p* < 0.001) and non-autistic neurodivergent (*p* < 0.001) participants. Neurotypical participants rated their experience of interviews as significantly more positive than non-autistic neurodivergent participants (*p* = 0.008). A Kruskal–Wallis test showed a significant group difference in the ratings of psychometric tests: χ^2^ = 29.79, *p* < 0.001. Post hoc Dunn-Bonferroni tests showed that neurotypical participants rated their experience of psychometric tests as significantly more positive than autistic (*p* < 0.001) and non-autistic neurodivergent (*p* = 0.008) participants.

**Table 3.** Autistic and non-autistic participants’ perceptions of recruitment methods.

	Autistic participants		Non-autistic neurodivergent participants		Neurotypical participants		Group comparisons
	No. of respondents	Mean score (*SD*)^a^	No. of respondents	Mean score (*SD*)^a^	No. of respondents	Mean score (*SD*)^a^	
Interview	212	2.21 (0.78)	63	2.75 (0.88)	83	3.13 (0.68)	A < NT*, ND*; ND < NT**
Written questionnaire	124	2.62 (0.84)	34	2.44 (0.75)	31	2.81 (0.75)	A = ND = NT
Online test^b^	112	2.59 (0.85)	36	2.39 (0.90)	48	2.77 (0.59)	A = ND = NT
Group task	108	1.60 (0.80)	32	2.53 (0.98)	43	2.67 (0.81)	A < NT*, ND*
Psychometric test^b^	91	2.13 (0.91)	35	2.29 (0.83)	55	2.96 (0.72)	A*, ND* < NT
Work trial	78	2.60 (0.98)	21	2.67 (0.97)	16	3.19 (0.66)	A = ND = NT
Skills assessment	21	2.85 (1.14)	1	4.00 (N/A)	3	3.33 (0.58)	A = ND = NT
Presentation	8	2.89 (0.93)	2	3.50 (0.71)	2	3.00 (0.00)	A = ND = NT

a Participants rated their perception of each recruitment method on a Likert-type scale from 1 (*very negative*) to 4 (*very positive*).

b Comparisons using Kruskal–Wallis tests, as the assumption for equal variance was violated.

* *p* < 0.001, **p = 0.008.

#### Providing feedback on recruitment processes

In total, 212 autistic participants, 41 non-autistic neurodivergent participants and 64 neurotypical participants responded to the question ‘has it been possible to provide feedback about your experiences of recruitment to employers?’^6^ The majority of autistic (*n* = 185, 87.3%), non-autistic neurodivergent (*n* = 33, 80.4%) and neurotypical participants (*n* = 47, 73.4%) reported that they had been unable to provide feedback to employers about their experiences (see Table 4). A chi-square test of independence indicated an overall significant association between neurotype and ability to provide feedback on recruitment experiences: χ^2^(2, *n* = 327) = 7.24, *p* = 0.027. Post hoc comparisons, adjusted using a Bonferroni correction, showed that significantly fewer autistic participants were able to provide feedback on their recruitment experiences, relative to neurotypical participants: χ^2^(1, *n* = 276) = 7.01, *p* = 0.008. There were no significant differences between autistic and non-autistic neurodivergent (χ^2^(1, *n* = 263) = 1.61, *p* = 0.205) or non-autistic neurodivergent and neurotypical (χ^2^(1, *n* = 115) = 0.76, *p* = 0.382) participants’ experiences.

**Table 4.** Experiences of providing feedback to employers about their recruitment processes.

	Autistic participants (*n* = 212)	Non-autistic neurodivergent participants (*n* = 51)	Neurotypical participants (*n* = 64)
**No, I have not been able to provide feedback to employers**	**185 (87.3%)**	**41 (80.4%)**	**47 (73.4%)**
I have not been able to provide feedback	183 (86.3%)	41 (80.4%)	46 (71.9%)
I have tried to provide feedback, but I was not listened to	2 (0.9%)	0 (0.0%)	1 (1.6%)
**Yes, I have been able to provide feedback about my recruitment experiences**	**27 (12.7%)**	**10 (19.6%)**	**17 (26.6%)**
Yes, I was asked by the employer to provide feedback	8 (3.8%)	7 (13.7%)	13 (20.3%)
Yes, but I had to ask the employer if I could provide feedback	12 (5.7%)	2 (3.9%)	4 (6.3%)
Yes, I have provided informal feedback	3 (1.4%)	0 (0.0%)	0 (0.0%)
I have given feedback to a recruitment agency	2 (0.9%)	1 (2.0%)	0 (0.0%)
Somebody else has provided feedback on my behalf	1 (0.5%)	0 (0.0%)	0 (0.0%)

Bolded items represent the superordinate response category.

### Qualitative results

**Figure 1.**
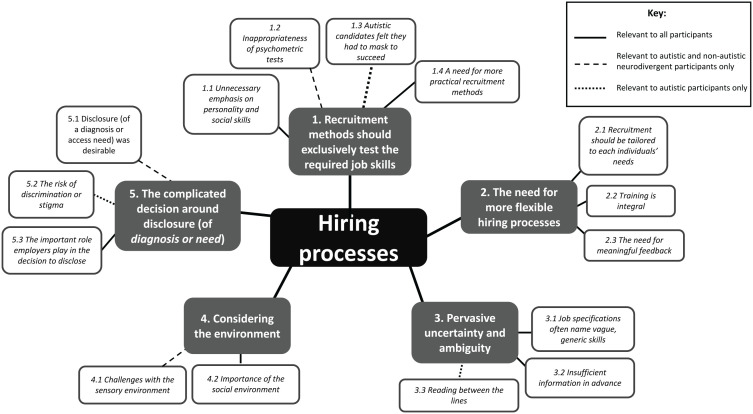
Thematic map of participants’ experiences of hiring processes.

We identified five themes from the open questions in which participants were asked to comment on particularly positive or negative experiences of recruitment, and provide suggestions for how processes could be improved. Most themes and subthemes were common to all three groups. As such, we report the findings across the whole sample. Any differences between the three groups are noted in the text and on the thematic map (see Figure 1). Illustrative quotes are accompanied by an ID that represents whether the participant is autistic (A), non-autistic neurodivergent (ND) or neurotypical (NT). Further supporting quotations can be found in Supplementary Table 2.

#### Recruitment methods should exclusively test the required job skills

Participants in all three groups noted that traditional recruitment methods place an *unnecessary emphasis on personality and social skills*, as opposed to the skills required for the prospective job role: ‘I feel that interviews are only a test of your acting and social skills’ (A-138). Participants reported finding the social aspects of recruitment particularly challenging, for example, finding it ‘very difficult to ‘sell myself’ (ND-040) in one-to-one interviews, or struggling to effectively contribute during group interviews and tasks: ‘[group tasks are] not good when you’re autistic, [I] don’t know when to interject, [and I] take a while to process information. . . that said, I think it’s a pretty horrible thing to do if you’re not autistic!’ (A-136). Indeed, one neurotypical participant reflected that ‘interviews are stressful and anxiety inducing’ (NT-007). In particular, participants were frustrated with the reliance on social skills recruitment methodology when social skills were not considered (by the candidate) to be an integral part of the prospective job: ‘The recruitment process and methods should reflect the role – if the job won’t include time-limited groups exercise with a presentation at the end of it, then why use this in the recruitment process?’ (A-127). Similarly, many autistic and neurodivergent, but not neurotypical, participants commented on the *inappropriateness of psychometric tests*. For example, some argued that ‘psychometric tests test social skills not peoples ability to do the job’ (A-162), while others felt they ‘measure my personality type’ (A-139) and might be particularly biased against autistic people: ‘psychometric tests are perfected specifically to filter out autistic traits’ (A-188). Participants expressed particular frustration that such tests would often be used at the beginning of the hiring process, meaning candidates who found psychometric tests challenging were screened out before they could show their potential:

I believe the online tests were an unnecessary screening process to filter out people without actually looking at your CV and professional merit . . . only after completing a literacy, numeracy and psychometric test was I actually able to speak to a real person. (ND-041)

The focus on social skills during recruitment meant that many *autistic candidates felt they had to mask to succeed*. Indeed, positive experiences of the interview process were typically reported when individuals were able to mask successfully (‘All the positive processes are the one(s) where I successfully masked and got through. Where I outsmarted them’; A-188) or rote learn responses they perceived as desirable: ‘I learned to interview very well because I learned to predict the type of questions asked. . . I had a prepared script. I could put on a perfect performance for the duration of the interview’ (A-048). Those who felt unable to mask effectively reported struggling to gain employment.

Linked to their negative experiences of recruitment methods that place emphasis on social skills, participants in all three groups expressed a need for employers to develop an ‘understanding that a traditional interview is not always the best way of assessing someone’s competence to do the particular job you are recruiting for!’ (NT-011). Relatedly, autistic, neurodivergent and neurotypical participants highlighted *a need for more practical recruitment methods*: ‘I would like to see more work trial or work exercise style interviews where people are put in realistic work situations. . . I feel that’s the only way to assess how someone could manage the role’ (ND-20). Indeed, those who had experienced more practical hiring techniques reported more positive experiences: ‘Work trials are positive interview experiences as I am confident in my abilities to carry out work-based tasks, they are less social and more practical so I can show my skills before my personality’ (A-167).

#### The need for more flexible hiring processes

Participants in all three groups expressed a need for more flexibility in hiring processes, and felt that *recruitment should be tailored to each individual’s needs*: ‘[We need] more flexible approaches, tailored to individual strengths’ (ND-003). While some participants suggested ‘there should be different recruitment processes for people with disabilities’ (A-181), others highlighted that ‘there can’t be a one size fits all approach’ (NT-076). Indeed, participants’ individual recruitment preferences were nuanced. For example, some participants felt online interviews would be beneficial (‘I would like to see autistic people given the chance to do interviews (if they are needed) by remote tech’; A-182) while others found them more challenging: ‘I recently did an interview over Skype. It was the worst thing imaginable’ (A-008).

Autistic, neurodivergent and neurotypical participants felt that *training is integral* in developing more flexible and inclusive hiring processes: ‘better training/understanding within those involved in recruitment (is needed)’ (NT-065). Autistic participants expressed a need for autism-specific training – on ‘the benefits of autism (to employers)’ (A-162), ‘how recruitment interviews impact on autistic applicants’ (A-082) and ‘knowledge and understanding about autism’ (A-006) more generally. Both autistic and non-autistic participants felt more holistic training on inclusive recruitment was required: ‘better training/ understanding within those involved in recruitment [is required]’ (NT-066). Specific suggestions included training on ‘disability awareness. . . and the benefit of differently abled employees’ (A-034), ‘unconscious bias’ (ND-061) and ‘more inclusive approaches to recruitment’ (NT-003). In particular, participants felt employers required more ‘education (*preferably [delivered] by autistic people*) about the types of adjustments which are helpful’ (A-005), so that they could be proactive in their approach to providing adjustments during recruitment: ‘employers should be more proactive with autistic applicants in offering reasonable adjustments, describing potential types of adjustment they can apply, and then actually applying them’ (A-012).

The need for tailored processes extended beyond the traditional interview, with participants in all three groups also expressing *the need for meaningful feedback* following unsuccessful recruitment. Indeed, many participants reported not hearing back from prospective employers following their application: ‘You rarely get even a “thanks for taking the time to see us” let alone any form of “we’re sorry but we felt that someone else was better for the job”’ (A-038) while others received standardised, generic feedback: ‘Where feedback is provided it is generally, “there were more suitable applicants”. This tells you nothing about YOUR reasons for not progressing further’ (A-224). As such, participants felt it was important to receive tailored, timely feedback to help them improve.

#### Pervasive uncertainty and ambiguity

Autistic, neurodivergent and neurotypical participants discussed the undue levels of uncertainty and ambiguity that pervade in all aspects of the recruitment process. Indeed, ambiguity was felt to be an issue right from the start of the hiring process with many participants noting that *job specifications often name vague, generic skills*: ‘We need to change role descriptions. We need to be more realistic about what we are recruiting a person to do. (i.e. do they really need to be adaptable, able to be a good consultant?)’ (NT-019). As a result, some autistic participants were not sure they possessed the necessary skills to apply: ‘I find it difficult to look for jobs and to know whether I would be suitable for the role’ (A-118). This ambiguity and resulting uncertainty was highlighted in further aspects of the traditional hiring process and typically fell into two main categories: (1) a lack of information in advance and (2) the need to read between the lines.

First, on a practical level, participants in all three groups often felt they were given *insufficient information in advance* regarding the hiring process. Indeed, examples of particularly negative experiences often involved unexpected situations or events during recruitment: ‘My worst experience was a group interview where we had to complete surprise group tasks. It was very overwhelming and I had to leave half way through’ (A-046). As a result, many participants expressed a need for employers to be clear in what candidates should expect from the hiring process: ‘[I would like] enough information to help reduce the “unknowns” (and cognitive load) on interview day: photos of building and interview room; names and photos of interviewers; length and basic content of interview and tests’ (A-042).

Second, autistic participants felt interview questions were often ambiguous and required a level of *reading between the lines*. For example, some participants spoke of their challenges in ‘understanding the meaning of the questions asked, and working out what response is wanted’ (A-085) and ‘answering “what would you do in situation X?” type questions [because] I can’t explain how I would react unless it actually happens’ (A-075). Suggestions for improvements in this regard included providing ‘interview questions in advance’ (A-161) and reducing the overall ambiguity of questions: ‘Questions should be to the point – none of this reading between the lines malarky’ (A-063).

#### Considering the environment

Autistic and non-autistic neurodivergent participants noted the importance of the physical environment in reducing anxiety during the hiring process. For example, participants reported experiencing *challenges with the sensory environment* involved in recruitment, including interviews that were often perceived to be ‘undertaken in “hostile environment” of bright lights, noise, whispering, circulating assessors’ (A-136). Indeed, particular challenges were noted in relation to artificial lighting, unregulated room temperatures, close proximity to others and the tactile experience of workwear. As such, participants felt it was important for employers to consider these factors and, where possible, make relevant adjustments to the sensory environment: ‘[Employers should] offer seating options and make it easy to be particular without feeling difficult. Cool room, no bright lights’ (A-219). Alternatively, suggestions were made for conducting interviews in settings that are familiar, and thus more comfortable, for candidates: ‘I prefer online interview as this removes environmental stressors allowing me to focus on my answers and take my time without appearing hesitant’ (A-167).

Similarly, participants in all three groups emphasised the *importance of the social environment* during recruitment. Positive experiences in this regard were often when the interviewer made an effort to create positive connections with the interviewee. For example, one participant reflected:

The simple act of an interviewer at a much better interview placing a jug of water and a glass on a little table next to me helped to calm me. Before the interview started the lead interviewer explained that they understood the process could be worrying, that I could ask them to repeat questions any time, that if I wanted a short break that was absolutely fine . . . I was offered the job but I would have felt good about the interview, the panel, and myself even if I had not been offered the job. (NT-008)

#### The complicated decision around disclosure (of diagnosis or need)

For many autistic and otherwise neurodivergent participants, *disclosure (of a diagnosis or access need) was desirable* – and, in many cases, was linked to a need for adjustments during the hiring process: ‘Ideally, a prospective employee feels safe enough to disclose before recruitment process. This would lead to the recruiting person acommodating the process accordingly’ (A-179). Despite being desirable, some autistic participants highlighted *the risk of discrimination or stigma* following disclosure: ‘[employers] only use [a diagnosis] as an excuse to discriminate against a person and not hire them’ (A-171). Indeed, many autistic, neurodivergent and neurotypical participants acknowledged *the important role employers play in the decision to disclose*: ‘[opportunities to disclose should] be done in a way that reassured the candidate that if they disclose a diagnosis, this absolutely would not impact whether they were offered the role or not’ (A-202). Suggestions for ways that employers could support candidates in their decision to disclose included taking ‘an overtly enabling approach that clearly states how the process has been constructed to be inclusive/adaptable’ (A-063), ‘more inclusive adverts [to] appeal to a wider audience’ (NT-113) and providing a list of adjustments that could be requested during the recruitment process: ‘Better advertising as part of the [hiring] process of the adjustments available’ (A-189).

## Discussion

The aim of the current study was to understand the unique hiring experiences of autistic candidates in the United Kingdom relative to the experiences of non-autistic neurodivergent and neurotypical candidates. Our results indicate clear distinctions between how autistic and non-autistic people endorse workplace recruitment methods. Specifically, autistic participants in this study reported having less positive experiences of interviews and group tasks (which favour social interaction) than non-autistic neurodivergent and neurotypical participants. Similarly, autistic and non-autistic neurodivergent participants had less positive experiences of psychometric tests (which are often perceived to be abstracted from the related job requirements) than neurotypical participants. Research suggests that those in senior-level positions may be offered psychometric tests more frequently than those in lower-level positions, both during initial recruitment and as part of the career development process (Harper, 2008). As such, the overrepresentation of neurotypical participants at a senior-level in this study could have influenced our findings. Indeed, a higher proportion of our neurotypical participants reported taking part in psychometric tests during the hiring process than our other groups. It is therefore possible that the more favourable rating of psychometric tests by neurotypical participants reflects a higher familiarity with this recruitment technique. Nonetheless, participants in all three groups highlighted the potential positive ramifications of using more practical recruitment methods that allow candidates to demonstrate their relevant skills and abilities, such as working interviews and skills assessments.

Despite highlighting differences in the endorsement of recruitment methods, the comparative approach of this study also allowed us to identify many commonalities in the qualitative experiences of recruitment across all three groups. Importantly, autistic, non-autistic neurodivergent and neurotypical participants reported challenges and, to some degree, frustration with the emphasis on social skills in traditional recruitment methods. Instead, our participants argued that the focus of recruitment should be on the key skills that are integral for the prospective job role. While such challenges with the social components of recruitment have previously been identified as a challenge for autistic people (e.g. Vincent, 2020), the unique comparative nature of our study allows us to highlight the similar challenges that both non-autistic neurodivergent and neurotypical job seekers also face. Indeed, participants in this study indicated that adjustments to the hiring process, including a reduced emphasis on social skills, could improve the experiences of *all* candidates.

We also demonstrated other similarities in our autistic, non-autistic neurodivergent and neurotypical participants’ views and experiences, including (1) the perceived need for more flexible recruitment methods; (2) a desire for more clarity surrounding the hiring process and what to expect and (3) the importance of the social envrionment during recruitment. Our autistic and neurodivergent participants also highlighted the important role employers play in one’s decision to disclose. The similarities between the recruitment experiences of autistic, non-autistic neurodivergent and neurotypical participants in this study highlight the need to be cautious of pathologising autistic people’s experiences of hiring processes. Indeed, the overlap in the experiences of autistic and non-autistic people is indicative of broader, systemic issues related to traditional hiring processes; changes that seek to mitigate such issues are therefore likely to benefit many rather than a few.

Nonetheless, there were a series of considerations and recruitment barriers that were specific to autistic adults in this study. First, while all participants negatively endorsed the emphasis of social skills during recruitment, only autistic participants reported that they had to mask their authentic self to successfully gain employment. This may be reflective of the learnt or natural tendency of neurotypical individuals to adapt their social performance to specific situations (e.g. using a professional language and demeanour in an interview). Consequently, non-autistic participants may not have discussed their engagement in masking within the survey as it is perceived as a ‘known unwritten social rule’. However, evidence shows that employers do show a preference towards employing non-autistic over autistic candidates (Ameri et al., 2018; Flower, Dickens & Hedley, 2019) and autistic people often experience stigmatisation and discrimination in the workplace, related to their autistic identity and traits (Johnson & Joshi, 2016; Müller et al., 2003). As such, it is possible that the degree to which autistic people feel they must mask, and the specific traits or behaviours they feel they must mask, differs to that of non-autistic people. That is, it is possible that autistic people must engage in a higher degree of masking, and must mask more aspects of their identity than non-autistic people, in an employment context. Yet, research demonstrates the detrimental effect masking can have on autistic individuals’ mental health and well-being (Bradley et al., 2021; Livingston et al., 2019; Pryke-Hobbes et al., under review; Raymaker et al., 2020; Tierney et al., 2016). As such, employers must do better to ensure autistic candidates are not discriminated against during recruitment. This may include improvements in the education and training those involved in recruitment receive surrounding autism specifically, and ‘hidden disabilities’ more generally. Indeed, existing research suggests that those involved in recruitment possess inadequate autism understanding (e.g. López & Keenan, 2014) and both autistic and non-autistic participants in the current study indicated a need for improvements in disability and inclusion training related to the hiring process. Specific suggestions of training programmes provided by the participants in this study included training concerning the benefits of a diverse workforce and training on providing adjustments during recruitment.

Second, autistic, but not non-autistic neurodivergent or neurotypical participants, reflected on the challenges they faced in ‘reading between the lines’ and decoding what employers were asking. Existing literature points towards the fact that autistic people can interpret language more literally than non-autistic people (e.g. Walenski et al., 2006) and autistic people often report requiring additional processing time to make sense of what is being asked of them (e.g. Honeybourne, 2019). Indeed, this is in line with research that indicates ambiguity in recruitment is a particular barrier for autistic people (Markel & Elia, 2016; Nagib & Wilton, 2020; Vincent, 2020). Yet, non-autistic neurodivergent *and* neurotypical people in the current study also experienced challenges related to ambiguity and uncertainty in recruitment. For example, participants in all three groups highlighted a need for improved clarity in the information given to candidates regarding the hiring process. Suggestions for improvements, made by our diverse range of participants, included (1) removing reference to non-essential skills in job descriptions; (2) making interview questions less ambiguous (cf. Maras et al., 2021) (3) providing clear documentation regarding the tasks that candidates will complete, the time the hiring process will take, and who will be involved and (4) providing clear deadlines regarding when candidates will hear the outcome of the hiring process. Indeed, our participants indicated that employers should feel confident that making such adjustments during recruitment could improve the experiences of autistic, non-autistic neurodivergent and neurotypical candidates alike.

Third, while participants in all three groups spoke of the role of the social environment during the hiring process, autistic and non-autistic neurodivergent people also reported experiencing unique challenges related to the sensory environment during recruitment. This is perhaps unsurprising given that many autistic people experience sensory sensitivities (Crane et al., 2009; Tavassoli et al., 2014). Indeed, a recent study in the United Kingdom involving interviews with 21 autistic young people indicated that the sensory environment during recruitment and employment can lead to sensory overload and psychological distress (Vincent, 2020). As such, and in line with the recommendations from participants in this study, employers should ensure they they have open communication with prospective employees about their needs – including sensory needs – and make adjustments where possible. For example, as suggested by our participants, ensuring that interviews are conducted in naturally lit, quiet rooms with regulated temperature and a neutral odour.

Finally, despite reflecting that disclosure of a diagnosis was desirable and may afford appropriate adjustments to the hiring process, autistic people had unique concerns regarding the potential stigma and discrimination associated with disclosing their autism diagnosis. This is consistent with existing literature which suggests that autistic people often experience concerns related to disclosing their autism diagnosis to others (e.g. Romualdez, Heasman et al., 2021; Sarrett, 2017; Vincent, 2020). This concern is not unfounded: research suggests that, while disclosure has been linked to improved access to employment for some autistic people (Ohl et al., 2017), stigmatisation and discrimination are not uncommon experiences following the disclosure of an autism diagnosis (Romualdez, Walker & Remington, 2021; Thompson-Hodgetts et al., 2020). Participants in this study highlighted a need for organisations to develop meaningful diversity and inclusion policies that harbour a diverse range of talent and make autistic people and non-autistic neurodivergent people more broadly, feel comfortable to disclose their diagnosis, should they wish to. For example, having a clear strategy for recruiting autistic and neurodivergent people and providing a list of adjustments that can be made during the hiring process, should candidates require them. A truly inclusive hiring process, however, may not require an individual to have, or to disclose, a specific diagnosis to access a hiring process that supports them. Indeed, neurotypical participants in this study reported experiencing a similar range of challenges to neurodivergent participants. As such, organisations should endeavour to proactively provide all candidates, regardless of whether they disclose a diagnosis, with adjustments during recruitment.

### Limitations

This research is not without its limitations. First, our sample may not be representative of the UK population. Indeed, most of our participants were well educated, in full-time paid employment and were of a white ethnic background. As such, this research only represents the recruitment experiences of a sub-group of the autistic, neurodivergent and neurotypical population. The lack of diversity in regard to ethnicity is particularly notable given that individuals from minority ethnic backgrounds often face unique barriers concerning recruitment (Cocchiara et al., 2016; Shore et al., 2021; Zschirnt & Ruedin, 2016). Indeed, this may mean that Black, Asian and ethnic minority autistic and otherwise neurodivergent people are multiply disadvantaged in regard to recruitment. Future research must redress this imbalance by developing meaningful relationships with minority ethnic communities and purposively recruiting autistic people from minority groups. The level of employment within our autistic group was also notable. While current estimates suggest as few as 22% of autistic adults in the United Kingdom are in paid employment (Office for National Statistics, 2021), more than two thirds of autistic participants in the current study reported being in full-time, part-time or self-employment. This difference is likely to be reflective of the fact that our research concerns employment experiences, which may have discouraged those who were not employed from participating. It is also important to note that all participants were able to complete a somewhat extensive survey regarding their recruitment experiences. As such, our results are unlikely to be representative of the recruitment experiences of people with intellectual disabilities or people who use non-traditional forms of communication. Similarly, the gender distribution in our autistic sample (more women than men), but not in our non-autistic neurodivergent or neurotypical samples goes against current estimates suggesting a 3:1 male: female ratio in relation to autism diagnosis (Loomes et al., 2017). Although this is not unusual for survey-based research like this one (see for example, (Arnold et al., 2019; Kapp et al., 2013), it may have influenced the pattern of findings reported herein.

Another potential limitation of the current study is that we did not seek to explore differences based on specific areas of neurodivergence or diagnoses, and instead divided the sample into autistic participants, non-autistic neurodivergent participants and neurotypical participants. The grouping of participants in this way was not intended to suggest that being autistic is distinguished from other forms of neurodiversity. Nor was it intended to suggest that neurodivergent people can, or should, be conceptualised as one homogeneous group. However, by dividing the sample in this way, we were able to highlight the (dis)similarities between autistic, non-autistic neurodivergent and neurotypical people’s experiences of hiring processes and highlight valuable questions for future research.

A third limitation is that we did not ask participants whether they had engaged in any form of supported employment, that is, schemes that typically involve teaching key workplace skills to support people through the hiring process and beyond (García-Villamisar & Hughes, 2007). Without knowing whether any of our participants had received any form of supported employment, it is difficult to determine if their perceived recruitment success could have been due to these supports. In any case, our participants highlighted several areas of the traditional hiring process that could be adjusted to improve the recruitment experiences of a diverse range of individuals across the United Kingdom, regardless of whether they receive employment support.

Finally, our recruitment methods might have influenced the findings. Some participants were recruited through corporate partners that were interested in the DARE project and therefore have an existing interest in autism, employment and making their employment processes more inclusive. As such, it is possible that people in the current study might have had more positive experiences of recruitment than the wider population. However, as our sample were self-selecting, it is also possible that our participants may have opted to take part as they had particularly negative experiences of recruitment. Nonetheless, we were able to highlight a series of meaningful barriers in recruitment for both autistic and non-autistic people, that employers should seek to address.

## Conclusion

This study demonstrated the unique recruitment experiences of autistic adults in the United Kingdom. While there were qualitative similarities in experiences, autistic people appeared to face a set of unique barriers to successful recruitment, over and above those that non-autistic neurodivergent and neurotypical people faced. While all participants reported being frustrated by the perceived unnecessary emphasis on social skills and personality traits in traditional hiring processes, only autistic candidates reported that they had to mask their authentic self to successfully gain employment. Similarly, despite the majority of participants reporting that disclosure of a diagnosis or access need was desirable, autistic participants expressed unique concerns surrounding the potential stigma and discrimination associated with their diagnosis. Employers have a critical role to play in reducing such inequalities in recruitment experiences. By actively offering and implementing adjustments for *all* candidates, employers can be confident that they are not only supporting their autistic candidates but also that the experiences of otherwise neurodivergent and neurotypical candidates are also likely to improve. Indeed, by developing more inclusive and accessible hiring processes, organisations can ensure that they see the best version of each candidate and do not overlook highly valuable talent.

## Supplemental Material

sj-docx-1-aut-10.1177_13623613221145377 – Supplemental material for Access to employment: A comparison of autistic, neurodivergent and neurotypical adults’ experiences of hiring processes in the United Kingdom Click here for additional data file. Supplemental material, sj-docx-1-aut-10.1177_13623613221145377 for Access to employment: A comparison of autistic, neurodivergent and neurotypical adults’ experiences of hiring processes in the United Kingdom by Jade Davies, Brett Heasman, Adam Livesey, Amy Walker, Elizabeth Pellicano and Anna Remington in Autism

sj-docx-2-aut-10.1177_13623613221145377 – Supplemental material for Access to employment: A comparison of autistic, neurodivergent and neurotypical adults’ experiences of hiring processes in the United Kingdom Click here for additional data file. Supplemental material, sj-docx-2-aut-10.1177_13623613221145377 for Access to employment: A comparison of autistic, neurodivergent and neurotypical adults’ experiences of hiring processes in the United Kingdom by Jade Davies, Brett Heasman, Adam Livesey, Amy Walker, Elizabeth Pellicano and Anna Remington in Autism

## Notes

1. Neurodiversity relates to naturally occurring differences in individuals’ neurology (Dwyer, 2022; Ne’eman & Pellicano, 2022).

2. People who display a different neurology to the general population are considered neurodivergent. The term neurodivergent encompasses a range of diagnoses including autism, attention-deficit hyperactivity disorder (ADHD), dyslexia and dyspraxia (Walker, 2014).

3. People who display broadly the same neurology as the general population are considered neurotypical (Walker, 2014).

4. A small number of participants (*n* = 12 of 377, 3.2%) completed the survey during the initial COVID-19 lockdown, when businesses were forced to shut down and many moved to remote working and thus hiring processes. In recognition of the impact the lockdown may have had on recruitment experiences, we analysed the data first with these participants included and second with them excluded, to see if their inclusion had an impact on the findings. There was no impact of the inclusion of these participants on our overall findings.

5. Given the significant barriers that exist in relation to receiving a formal autism diagnosis, we chose not to exclude self-identified autistic people (Lai & Baron-Cohen, 2015). Nevertheless, we ran analyses to examine whether there were any differences in the demographic details between formally diagnosed autistic participants and those who self-identified as autistic. There were none. As such, all autistic participants were considered together.

6. It should be noted that participants were not asked whether they had wanted to give feedback, only whether they had been given the opportunity to do so.
