# Supplementary material for: Access to employment: A comparison of autistic, neurodivergent and neurotypical adults’ experiences of hiring processes in the United Kingdom
Source: Autism. 2023 Jan 4;27(6):1746–63. doi: 10.1177/13623613221145377 (PMC10375005; doi:10.1177/13623613221145377)
Supplement: sj-docx-1-aut-10.1177_13623613221145377 – Supplemental material for Access to employment: A comparison of autistic, neurodivergent and neurotypical adults’ experiences of hiring processes in the United Kingdom [file sj-docx-1-aut-10.1177_13623613221145377.docx]

**​Experiences of recruitment survey**

Recruitment processes include all the steps from a job being advertised to being informed about the outcome of the final assessment or interview.

**What type of recruitment processes have you experienced?**

|  | **Have you experienced this process?** | **How positively would you rate those experiences?** | | | |  |
| --- | --- | --- | --- | --- | --- | --- |
|  | *Tick if ‘yes’* | Very negative | Negative | Positive | Very positive | |
| Online test | **□** | ○ | ○ | ○ | ○ | |
| Psychometric test | **□** | ○ | ○ | ○ | ○ | |
| Interviewing | **□** | ○ | ○ | ○ | ○ | |
| Written questionnaire | **□** | ○ | ○ | ○ | ○ | |
| Work trial | **□** | ○ | ○ | ○ | ○ | |
| Group task | **□** | ○ | ○ | ○ | ○ | |
| Other (please specify)  ____________ | **□** | ○ | ○ | ○ | ○ | |
| Other (please specify)  ____________ | **□** | ○ | ○ | ○ | ○ | |

**Has it been possible for you to provide feedback about your experiences of recruitment to employers?**

○ Yes, I was asked by the employer to provide feedback about recruitment experiences

○ Yes, but I had to ask the employer if I could provide feedback

○ No, I have not been able to provide feedback to employers

○ Not applicable

○ Other (please specify) _______________________________________________________

**Please discuss any positive or negative recruitment processes that stand out in your experience:**

[open text box]

**What would you like to see in terms of improving practice around recruitment processes?**

[open text box]
