# Supplementary material for: Access to employment: A comparison of autistic, neurodivergent and neurotypical adults’ experiences of hiring processes in the United Kingdom
Source: Autism. 2023 Jan 4;27(6):1746–63. doi: 10.1177/13623613221145377 (PMC10375005; doi:10.1177/13623613221145377)
Supplement: sj-docx-2-aut-10.1177_13623613221145377 – Supplemental material for Access to employment: A comparison of autistic, neurodivergent and neurotypical adults’ experiences of hiring processes in the United Kingdom [file sj-docx-2-aut-10.1177_13623613221145377.docx]

| **Themes** | **Sub-themes** | **Illustrative Quotes** | | | |
| --- | --- | --- | --- | --- | --- |
|  |  | **Autistic** | | **Neurodivergent** | **Neurotypical** |
| 1. Recruitment methods should exclusively test the required job skills | *1.1 Unnecessary emphasis on personality and social skills* | “I feel that interviews are only a test of your acting and social skills”  “[negative experiences include] interviews where I need to perform social skills which have nothing to do with the work I would be doing if they gave me the job” | | “I find it very difficult to ‘sell myself’” | “Interviews can focus too much on personality rather than ability to do the job” |
|  |  | |  |  |  |
|  | *1.2 Inappropriateness of psychometric tests* | “psychometric tests test social skills not peoples ability to do the job”  “psychometric tests are perfected specifically to filter out autistic traits” | | “I believe the online tests were an unnecessary screening process to filter out people without actually looking at your CV and professional merit”  “[there should be] no more psychometric [tests]” | N/A |
|  | *1.3 Autistic candidates felt they had to mask to succeed* | “I have never not masked throughout the recruitment process; I believe I would never be employed if I didn’t mask” | | N/A | N/A |
|  | *1.4 A need for more practical recruitment methods* | “Any job where I have had the opportunity to show what I am capable of rather than just sit through an interview has always been better.”  “more places should do trail shifts. Then they get to see me work and evaluate me on that rather than on my terrible social skills.” | | “I would like to see more work trial or work exercise style interviews where people are put in realistic work situations (i.e., team meetings, individual work, etc) as I feel that’s the only way to assess how someone could manage the role.”  “more work trials - actually showing you can do the job when the pressure is taken out of the situation” | “[There needs to be] an understanding that a traditional interview is not always the best way of assessing someone's competence to do the particular job you are recruiting for!” |
| 2. The need for more flexible hiring processes | *2.1 Recruitment should be tailored to each individual’s needs* | “Asking people what they need to make the interview work best for them [would be beneficial]” | | “[We need] more flexible approaches, tailored to individual strengths” | “there can't be a 'one size fits all' approach.” |
|  | *2.2 Training is integral* | “In an ideal world, employers would have sufficient knowledge of autism not to reject autistic people for displaying traits such as lack of eye contact or social awkwardness”  “Education (preferably by autistic people) about the types of adjustments which are helpful” | | “Unconscious bias and conscious inclusion training should be compulsory for all recruiters.” | “Better training/ understanding within those involved in recruitment [is needed]” |
|  | *2.3 The need for meaningful feedback* | “Where feedback is provided it is generally, [it is like] “there were more suitable applicants”. This tells you nothing about YOUR reasons for not progressing further” | | “The whole process was very drawn out with long periods where no feedback was received” | “negative [experiences include] not getting feedback [or] being left waiting for a long time” |
| 3. Pervasive uncertainty and ambiguity | *3.1 Job specifications often name vague, generic skills* | “Every job description no matter how menial the actual job is quotes "excellent communication skills” | | “Services [are] needed to get round the prevalence of job listings asking for communication skills.” | “We need to change role descriptions. We need to be more realistic about what we are recruiting a person to do. (i.e., do they really need to be adaptable, able to be a good consultant?)” |
|  | *3.2 Insufficient information in advance* | “My worst experience was a group interview where we had to complete surprise group tasks. It was very overwhelming and I had to leave half way through.” | | “Applicants are not treated very well in the early stages [of recruitment]. If someone has made the effort to apply for a job they should be treated with respect and given accurate information about the stages involved in the recruitment process (e.g. phone interview, face-to-face interview) and the timescales for these stages.” | The only [negative] thing that really stands out is one role where I didn't know anything about it until I was in the interview, which didn't help.” |
|  | *3.3 Reading between the lines* | “Use of "what would you do in situation X " type questions [are challenging]. I have difficulty in applying something I have done in the past to a theoretical situation.” | | N/A | N/A |
|  |  | |  |  |  |
|  |  | |  |  |  |
| 4. Considering the environment | *4.1 Challenges with the sensory environment* | “Wearing formal work wear is uncomfortable and restricting”  “[Interviews are] undertaken in 'hostile environment' of bright lights, noise, whispering, circulating assessors.” | | “Sensory issues make all work dress codes wrong” | N/A |
|  | *4.2 Importance of social environment* | “My interview with [organisation] was a relaxed chat with a Chief Engineer over coffee. That was the best experience I've had.” | | “Successful [interviews] have felt more conversational, than formal questioning.” | “The simple act of an interviewer at a much better interview placing a jug of water and a glass on a little table next to me helped to calm me ... I was offered the job but I would have felt good about the interview, the panel,and myself even if I had not been offered the job.” |
| 5. The complicated decision around disclosure (of diagnosis or need) | *5.1 Disclosure (of a diagnosis or access need) was desirable* | “Ideally, a prospective employee feels safe enough to disclose before recruitment process. This would lead to the recruiting person acommodating the process accordingly.” | | “Being made comfortable to disclose the diagnosis [is important]” | N/A |
|  | *5.2 The risk of discrimination or stigma* | “[employers] only use [a diagnosis] as an excuse to discriminate against a person and not hire them” | | N/A | N/A |
|  | *5.3 The important role employers play in the decision to disclose* | “Better advertising as part of the process of the adjustments available to candidates [would be beneficial]”  “[opportunities to disclose should] be done in a way that reassured the candidate that if they disclose a diagnosis, this absolutely would not impact whether they were offered the role or not.” | | “[It is important] for employers to openly welcome neurodiversity people and understand we are not all robots” | “More inclusive adverts i.e., appeal to a wider audience [would be beneficial]” |
